# Medieval re-creation and translation in Edwin Morgan and Derek Jarman’s archives: A dialogue

**DOI:** 10.1057/s41280-024-00319-0

**Published:** 2024-06-10

**Authors:** Francesca Brooks, E.K. Myerson

**Affiliations:** a Department of English and Related Literature, University of York, York, UK; b Emmanuel College, University of Cambridge, Cambridge, UK

## Abstract

This dialogue brings together two archives: the Derek Jarman collection at Tate Britain and the Edwin Morgan manuscripts at the University of Glasgow Library Archives and Special Collections. Both Jarman and Morgan were queer artists who reforged the medieval past through visual and verbal processes, including scrapbooking, collage, and translation. The dialogue also brings together two critics, Francesca Brooks and E.K. Myerson, who have been responding to these archives through a combination of traditional scholarship and creative practice. Both in their approach to the medieval past and in their multimedia, visio-verbal practices, Jarman and Morgan’s archives are generative, fluid, and expansive spaces that invite embodied, affective, and creative responses. The dialogue explores what it means to be a medievalist working in the modern archive and how our critical approaches to the medieval subjects of our research might be changed and transformed by this work.

## Francesca Brooks

In June 2022 we met at the Forgeries Workshop organised by Rebecca Menmuir and Hannah Armstrong. During our conversations at the workshop, we realised that there was a lot of common ground between the archival projects we were individually working on. What does it mean to be a medievalist working with the modern archive? What happens to medieval culture when we meet it through the eyes and imaginations of twentieth-century creative practitioners, as documented in the institutional and intimate space of the archive? How is the medieval past forged, or indeed reforged, by the hybrid creative practices of postmedieval artists and writers? While I have been working with the archives of the Scottish poet and translator Edwin Morgan (1920–2010) at the University of Glasgow Library, you have been working with those of the British artist, filmmaker, and activist, Derek Jarman (1942–1994) in the Tate Gallery Archive. Although the other pieces in this cluster consider forgeries in terms of deception, Jarman and Morgan’s works reveal a practice of forgery as creative self-exploration, articulated through the language and materials of the past as they encounter the present.

For Edwin Morgan, a translator of medieval poetry, the translation of a medieval text is not a forgery. In the introduction to his 1952 translation of *Beowulf*, Morgan asserts that in translation ‘[c]ommunication must take place; the nerves must sometimes tingle and the skin flush, as with original poetry’ (2001, xii). Although some translations of *Beowulf* strive to be forgeries and attempt to achieve a kind of ‘authenticity’—the scare-quotes are Morgan’s own—by making use of archaisms and artificial language, for Morgan these translations have kept *Beowulf* ‘faintly gleaming like a dragonfly under an inch of amber’ (2001, xv). *Beowulf* is preserved as a dead thing in these forged translations, but it can be reforged and brought to life if the writer makes ‘contact with the poetry’ of their own time (Morgan 2001, xiii). What was Jarman’s relationship to the idea of ‘forgery’?

### E.K. Myerson

For Jarman, forgeries were as worthy of veneration as authentic artefacts. In his diary in March 1990, he recalls visiting a British Museum exhibition of fakes in their collection, among which he found: ‘oh heavens, my little twelfth century pearl reliquary from Scotland is there, the most precious object in the museum. But it is so exquisite, being “fake” cannot diminish it’ (2018, 253). This reliquary was not a postmedieval forgery, but an instance of medieval devotion to fake relics. In both Jarman’s works and in medieval culture, the meaning of objects is created through communal practices. Forgeries become sacred through public, repeated rituals, that is: religious and/or queer ceremonies. In that sense, the medieval provides a model for Jarman’s artistic practice, as well as often being the subject of his own remaking.

### Brooks

In comparable although distinct ways, Jarman and Morgan used visio-verbal processes of scrapbooking, collage, and assemblage to reforge the medieval culture that was central to their imaginations. In picking up and developing those conversations from the summer of 2022, I wonder if you might begin by introducing the material you have been working with in the Derek Jarman archive and its relationship to medieval culture?

### Myerson

Thanks for this introduction—I’m really interested to see how these terms of scrapbooking and assemblage unfold in our conversation. I’ve looked at two main archival collections: Jarman’s project books for his films *The Garden* (1990), *Edward II* (1991), and *Blue* (1993), which are now held in the Tate Britain, and draft scripts relating to the unrealised medieval film project, *Bob-up-a-Down*, held at the British Film Institute (BFI). For this dialogue, the most relevant materials are those in the Tate collections, dated 1989–1994. During this period, Jarman returned to the medieval past to find an alternative language of queer martyrdom and healing, which he invoked in order to challenge hostile discourses around HIV/AIDS (Mills 2022; Myerson 2023a). In 1994, Jarman died from an AIDS-related condition. These archives are inseparable from the knowledge of Jarman’s death and the death of Keith Collins, Jarman’s partner and carer. Collins was the custodian of these materials until 2018, when he passed away from cancer and bequeathed the archives to the Tate repository. I’ve been reflecting on theories of mourning in relation to the archive, and especially the place of grief in queer archives, as a way of processing my own often quite traumatised response to some of these beautiful objects. The Jarman archives include ephemera which constitute a queer archive (Kumbier 2014), records of the AIDS crisis which demand their own approach (Cvetkovich 2003), and aesthetic texts and images, foregrounded through their position in a gallery. In my work, I tried not to resolve these distinct elements; I did not want to aestheticise the AIDS crisis by subsuming these histories into a narrative of Jarman as an individual artist. I aim to recognise the Jarman archives as plural, even within one repository (Myerson 2023b).

Jarman’s notebooks are often highly decorated in a manner which directly evokes medieval devotional traditions. For example, one small notebook, a project book for the film *Blue* (1993), dated July 1989, titled ‘The Blueprint for Bliss,’ has a hand-gilded cover, with a pebble affixed over the gold leaf; the frontispiece is painted International Yves Klein blue, with the title written in gold (London, Tate Britain Archives, TGA 20157/1/2). In the text, Jarman describes this blue as the space ‘where the music of the spheres drives the universal harmony,’ alluding to medieval ideas of the heavenly spheres, which were thought to chime as they rotated against one another. In 1989, Jarman had recently moved to Prospect Cottage, a fisherman’s cottage in Dungeness, Kent, in response to his HIV+ diagnosis (Peake 1999, 395). The transcendence of the medieval, aesthetic and spiritual, was liberating to Jarman as a queer artist, as a sick artist, and as an avant-garde historian.

In Jarman’s practice, the premodern is integrated with Jarman’s activism, in a very direct way. For example, in his film *Edward II* (1991), scenes of the life of the Plantagenet King are montaged with footage of an Outrage! demonstration. In the archives, I looked at a hand-gilded project book for this film marked ‘Edward II Dungeness 1990’ (London, Tate Britain Archives, TGA 20213, uncatalogued). The book contains typewritten scripts for Edward II, with a feather stuck into the inside front cover, and at the back, a photograph of Andrew Tiernan, who played Gaveston, naked and doing the washing up. The photograph is opposite a note from Keith Collins addressed to Derek Jarman, reading: ‘Fur Beast. Now I’ve given you my favourite shirt you must be sweet and furry always. H. Beast.’ Jarman’s scripts come to life in these domestic scenes—I can see him redrafting at a kitchen table with talented, sexy, younger men gathered around him, doing his dishes, and taking off their shirts. As multimedia artworks, Jarman’s project books create juxtapositions between texts, objects, and images, resulting in intimate, fragile documents.

Passing your own question back, could you reintroduce me to the materials which you’ve been working on in Morgan’s archives and remind me of their relationship to the medieval?

### Brooks

I am fascinated and moved by your account of mourning in the archive and how Jarman’s ongoing encounter with the Middle Ages is one of transcendence and liberation. There are so many threads to pick up here, and I want to come back to that idea of liberation to talk about Morgan’s sense of his scrapbooks as a safe, playful, and subversive space for exploring a queer identity that he was not yet ready to make public.

**Fig. 1:**
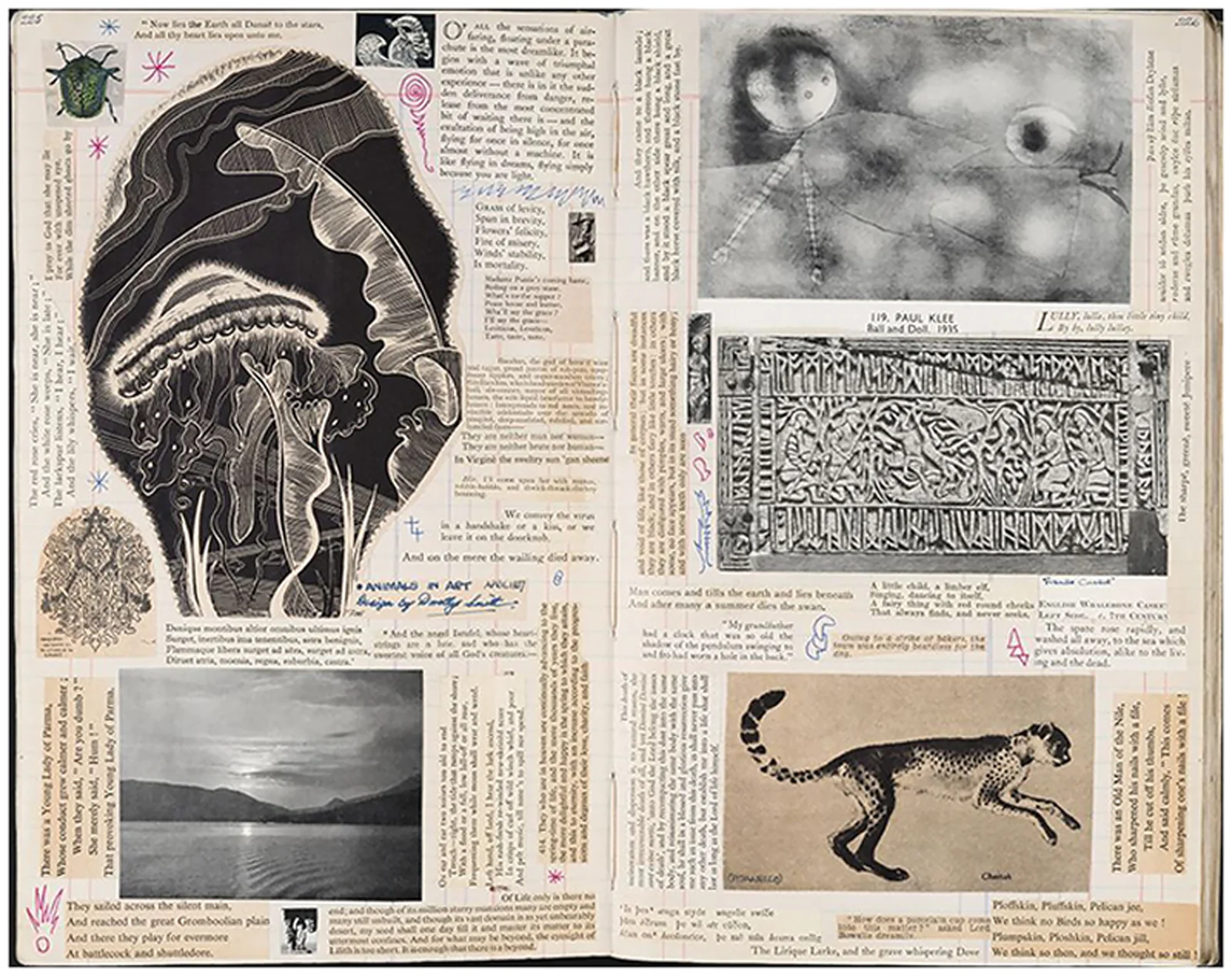
MS Morgan C/1, Page-spread from Scrapbook 1, 225–26, University of Glasgow Library Archives and Special Collections. Photo © University of Glasgow Library Archives and Special Collections. Courtesy of the Edwin Morgan Trust and the University of Glasgow Library.

As part of a short-term fellowship with the University of Glasgow Library Archives and Special Collections, I have been working with a collection of scrapbooks created by Edwin Morgan across four decades between 1931 and 1966. There are sixteen scrapbooks in the library collection, all marked up by Morgan with date ranges for their creation but continuously numbered across the volumes, running to 3600 pages in total. They are vast, unruly, and uniquely magnificent pieces of work that are testament to the incredible diversity and voraciousness of Morgan’s interests. In the first scrapbook alone, for example, quotations from Old English poems including *Beowulf, Maxims*, and *The Phoenix*, and images from early medieval artefacts and manuscripts, jostle for space alongside pictures of jellyfish, sea anemones, and goose barnacles, photographs of blasted snow-bound mountains, or surreptitious allusions to queer desire, as well as news items about cosmic rays and potential radio transmissions intercepted from outer space. In Fig. 1 you can see an example of this, where a print of a jellyfish faces an image of the early medieval Franks Casket (Glasgow, University of Glasgow Library Archives and Special Collections, MS Morgan C/1, 225–6). Morgan began the scrapbooks when he was a teenager, and they sustained him through early periods of experimentation, but the work fizzled out when his career as a poet and translator really began to take off. In this sense they are entangled, in fascinating ways, with Morgan’s first published book-length poem and first translation, *Beowulf*, published in 1952 by the Hand and Flower Press.

Chris Jones has written about the scrapbooks in relation to Morgan’s study of and play with Old English poetry (Jones 2006, 122–81; Jones 2012), but I wanted to think in quite a focused sense about how the visual process of scrapbooking might have informed the verbal work of translating *Beowulf*. One relationship that immediately occurred to me in the archive was thematic: the scrapbooks reveal an ongoing fascination with the strange and the unfathomable, whether that be the new threat of nuclear devastation, inexplicable materials unearthed from the past, or the wonders of the natural world. One scrapbook has a double page spread about slug courtship pasted in, and elsewhere decontextualised clippings have been incorporated: ‘out of the past come these ivory “pretzels” to baffle archaeologists,’ reads one (MS Morgan C/8). James McGonigal and Sarah Hepworth describe these mixed images as establishing ‘an expressive tension, where meaning struggles towards articulation’ (2012, 12). The Old English *Beowulf* is also interested in testing the limits of knowledge and marvelling at what is beyond our ken, from the unfathomable depths of the mere of the Grendelkin to emotions and instincts that cannot be expressed in language (Bolintineanu 2016; Saltzman 2018). A question that arises out of this comparison of visual and verbal might be how the baffling aspects of Morgan’s modernity, such as conflict, war, and nuclear devastation, reforge the *Beowulf* poem as he translates it. As Chris Jones writes, ‘Morgan prefers to see the medieval not only as having once been contemporary, but also as having once been futuristic’ (2006, 149).

Morgan wrote that the scrapbooks are ‘a mixture of autobiography, documentary, and art’ (MS Morgan 4582/56), but this kind of prismatic personal and historical reading can also be applied to *Beowulf*. In 2001 when Carcanet republished the translation, Morgan added a preface in which he revealed that ‘begun shortly after I came out of the army at the end of the Second World War, [*Beowulf*] was in a sense my unwritten war poem,’ and in his poem ‘Seven Decades,’ Morgan writes more suggestively of how he ‘translated Beowulf for want of love’ (2001, ix; 1990, 594). Morgan’s translation of a poem about homosocial relationships and kinship between men is reframed retrospectively as an expression of repressed desire. However, in the scrapbooks from the time Morgan is much more open about these potential queer connections. Bridget Moynihan has discussed ‘meta moments’ in the scrapbooks, where the cut-out words Morgan has arranged and reassembled appear to speak reflexively about the process of scrapbooking and collage (Moynihan 2022). There are also other kinds of ‘meta moments’ in the books: queer disclosures that speak of Morgan’s sexuality. Morgan did not come out publicly until the 1980s when the legalisation of homosexuality was extended to Scotland, but he is ‘out’ in the scrapbooks for those who might be attuned to his playful references. If *Beowulf* was a cloak or a ‘mask’ for Morgan’s desire (Jones 2006, 124), the scrapbooks offer a joyful release for his passions.

Perhaps we might stay a while with the sense of the queerness of the Middle Ages. What is it about medieval mysticism and medieval cultures of devotion that appeals to Jarman as a queer artist? I also want to ask more about your affective response to Jarman’s archive. Am I right in thinking that this was the first time you had worked with a modern rather than a medieval archive? How has working with such personal material from the recent past affected or changed your approach to the medieval material you work with?

### Myerson

I’ll begin with an aspect of medieval devotion which appealed to Jarman as a queer artist. In *Modern Nature*, Jarman writes: ‘The Middle Ages have formed the paradise of my imagination […]. It is […] something subterranean, like the seaweed and coral that floats in the arcades of a jewelled reliquary’ (2018, 208). This quotation appears at the start of Robert Mills’ pathbreaking book-length study on Jarman’s medievalism (2018), and so forms the foundations of the growing critical field of medievalist scholarship on Jarman, for good reason. The image articulates Jarman’s sense of the transcendent instability of the premodern. Unlike the present-day institutional church, which Jarman saw as a site of rigid repression and oppression, medieval Christianity offers a language beyond modern categories. Jarman’s image of the Middle Ages as a submerged reliquary was in my mind as I read your account of Morgan’s images of ‘jellyfish, sea anemones, and goose barnacles.’ It seems to me that both artists were drawn to the premodern past not as a fixed, contained world, but as a natural and strange region bordering our own—like the deep sea.

I find your language of the ‘unfathomable’ useful in articulating a critical discourse of the unexplorable. In both the Jarman collections and in medieval mystical texts, apophasis is essential. For Jarman, the unknowable element of medieval devotional works provided a tool of resistance to conservative notions of gender, sexuality, and the body.

You are right, this was the first time I had worked with a modern rather than a medieval archive—and it was a really transformative experience for me. I see Jarman as having what Carolyn Dinshaw has called ‘a queer medievalist’s touch’ (1999, 54), creating a transhistorical dialogue predicated on the urge to form communities across time.

Going back to the Manuscripts room at the British Library after spending time in the Tate collections, I began to find myself touched by late medieval plague treatises. Reading medieval cures annotated with Latin terms like ‘*nota*’ (‘note this’)—which I had previously found commonplace—I began to feel the traces of individual bodies, hands that came to these books with urgent desires for cures for often incurable illnesses. I don’t want to draw a direct parallel between premodern bubonic plague and the AIDS crisis—and I don’t think that Jarman invites us to do so. However, I was moved by the way in which Jarman’s experience of living through a pandemic made him a more empathetic reader of premodern medical treatises. I had not previously employed an affective methodology, but Jarman’s archives seemed to demand it, and I found that a first-person voice in my criticism became increasingly present. My own body in the archives became the acknowledged site in which my research was taking place—which I suppose had always been the case, but I hadn’t been aware of that previously.

I am struck by the sheer scale of the archives that you’ve been working with—3600 pages from juvenilia to later works! In a basic sense, how do you begin to approach these objects within the context of a short-term fellowship? I imagine that there is plenty in the Morgan archives that by necessity you have had to leave unstudied, for now. I wonder if your approach was influenced by Morgan’s own selective approach to the medieval past, which itself (like these archives) feels both vast and fragmentary. The image of *scrap*-booking is evocative of archival methodologies.

One obvious difference between Morgan and Jarman is in their public relationship to their sexuality. While Morgan was—understandably—private until decriminalisation, Jarman was known for his queer activism. That seems to alter the archive as a space. I am interested in your experience of the archive as a space of disclosure. I definitely experienced intimacy with Jarman in the archives—but there is a certain performative quality to Jarman’s intimacy with his readers: when he’s writing his diaries, he’s always intending to publish.

Could you tell me about Morgan’s own intentions for these scrap-books—did he aim to publish them within his lifetime?

### Brooks

I like your idea that the Middle Ages is something fluid rather than fixed for both Morgan and Jarman, ‘like the deep sea’ neighbouring our own terrestrial world. This week I have been reading some of Morgan’s translations, and I came across something that speaks to Jarman’s decadent image of the ‘subterranean’ or ‘submarine’ Middle Ages. In introducing the world of Eugenio Montale’s poems, which he had translated, Morgan writes:

Onto this harsh coast are thrown the cuttle-bones (‘ossi di sepia’) of another world: relics of a living creature, evidence from a mysterious, submarine, nearly inscrutable life: beautiful but strange objects, like the poems themselves. There is no cuneiform on these stark pelagic potsherds. How can we read them? What message is sent? (1996, 4)

Although Morgan is talking about twentieth-century Italian poetry, and not Old English, that sense of what is ‘nearly inscrutable’ but asks to be deciphered chimes with the partially intelligible lure of ‘alien’ messages of other kinds. Morgan encourages us to revel in the ‘beautiful and strange objects,’ rather than resisting any aestheticizing impulse. However, as your work with Jarman attests, there is a queer, creative, and generative joy in admiring the strange beauty of the past, which can transcend the aesthetic and become, as you write, ‘a tool of resistance.’ Morgan’s translations were always political in the sense of establishing and holding open lines of communication.

Morgan did want his scrapbooks published and pitched them to the literary agents Christy and Moore in the 1950s and to the editor at Carcanet, Michael Schmidt, in the 1980s (McGonigal and Hepworth 2012, 1). They do, therefore, also have a performative quality to them. Ellen Gruber Garvey argues, for example, that scrapbook makers engage in ‘performing archivalness [in] the will to save, organize, and transmit knowledge through a homemade archive’ (2013, 20; see Moynihan 2020, 3). Copyright surrounding the scrapbooks is incredibly complicated, however, and has remained a barrier to publication (Deazley et al. 2017; Moynihan and Putra 2019, 5). During my induction in the archive, I learnt that I would not be able to take any photographs of the scrapbook pages because of these copyright issues. How then should I record my encounter with these 3600 pages? My first response—largely one of disorientation—was to try to create a description of each of the pages I examined, but this was ultimately going to be an impossible labour. The question of copyright has been renewed for me as we have moved towards publishing this dialogue: every element of each individual scrapbook page, visual and verbal, potentially belongs to a different copyright holder. As you will see in Fig. 7, having been unable to secure copyright for Salvador Dali’s *Visage of War*, I have followed the model of the *Digitising Morgan* project’s ‘Risk Filter’ and blanked out this element of the scrapbook page (Deazley et al. 2017).

James McGonigal’s biography of Morgan provided me with a solution to this archival problem. McGonigal uses Morgan’s comments about the Arabic or Persian divan to suggest how in the scrapbooks ‘randomness [can be] a “structuring principle” ‘; he quotes Morgan who describes the divan as a collection of poems, ‘something that you enter; you move around; you can cast your eye here and there, you look, you pick, you perhaps retrace your steps’ (McGonigal 2010, 44–45). I took this as permission to find my own way through the divergent and multiplying interests of the scrapbooks. I started to keep much more focused notes primarily confined to the pages that were incorporating Old English or early medieval material, while also noting down observations about themes and connections that seemed to be emerging across the books. This was a negotiation of my own directed interests, and a response to what I was learning about Morgan’s own life and passions in the archive—a constant shift between micro and macro scales of attention.

I have been reading medieval literature through modernist archives since my PhD and have learnt that my own interests and approaches will always be slightly reconfigured by this encounter with the medieval past through the eyes of another (Brooks 2021). By their very nature the scrapbooks collapse, blur, and transgress the boundaries between categories of interest. As academics we are very invested in those boundaries because we specialise in niche areas that are determined by disciplinary affiliations and periodisation, but writers and artists do not feel a sense of those limits, and it was hard to bring them to the scrapbooks. Over the course of the fellowship, I found myself becoming more fascinated by how extra-terrestrial phenomena, or even the threat and fear of nuclear war, might interact with Morgan’s passion for ancient and medieval languages (Ferhatović 2016). Although I had not worked with queer theory before, this aspect of Morgan’s creative identity was impossible to overlook. In a slightly more unexpected turn, and perhaps in tune with your own embodied experience in the archive, I spent my Glasgow evenings making collaged scrapbooks of my own.

One thing I have been considering is whether an article on Edwin Morgan’s scrapbooks should itself be structured like a scrapbook page: moving between micro and macro scales to think about scrap/s, page, page-spread and scrapbook/s in ways that are discrete but interconnected and could plausibly be cut-up and rearranged. In my previous response I talked about a macro scale—a thematic sense of the scrapbooks as a whole—but one example of a micro approach might come from looking at how an individual line of Morgan’s *Beowulf* translation functions as a kind of verbal collage or assemblage. In Morgan’s *Beowulf*, for example, lines 111–13 read: ‘then awoke all evil creatures, ogres and elves and monsters, likewise giants/Kobolds and gogamagogs, lemurs and zombies, and the brood of the titans’ (Morgan 2001, 3–4). Morgan’s translation is fantastically weird, and I feel like I need a dictionary on hand to make sense of all the images—in Germanic mythology ‘kobolds’ are sprites that haunt houses and live underground, for example. The translation is analogous to the catalogue of beasts crafted across the scrapbooks (including the courting slugs), and they also have something in common with the multilingual wordlists Morgan kept, which are now in the Mitchell Library in Glasgow—a kind of verbal scrapbook or ‘word hoard’ to match his visio-verbal books (EM MORGAN [SCRAPBOOK, 1951–]).

Perhaps we should draw our conversation back to questions about ‘forging,’ ‘forgeries,’ ‘authenticity,’ and perhaps even ‘inauthenticity.’ How do you see these terms informing your work with Jarman’s archives?

### Myerson

I agree it would be good to refocus on the idea of ‘inauthenticity.’ I want to go back to the terminology of ‘re-forging’ with which our conversation began. In an article entitled: ‘The Year in “Re-.” ‘ Martha Buskirk, Amelia Jones, and Caroline A. Jones (2013) catalogued the relevant terms in the art world: ‘recollect,’ ‘reconstruct,’ ‘re-create,’ ‘reproduce,’ ‘relic,’ and so on. Alexander Nagel returned to that catalogue recently, commenting that ‘[r]eworking older materials’ has now been established as a central aspect of modern and contemporary art (2023, 9). Actually, ‘re-forging’ wasn’t on their list, so it would be a good term to add to a revised catalogue. I want to draw attention to our own ‘re-’ terms, by re-reading our conversation so far. So far, we’ve used: ‘relate’ (and cognates ‘relation’ and ‘relationship’), ‘reflecting,’ ‘repository,’ ‘response,’ ‘reintroduce,’ ‘remind,’ ‘reception,’ ‘republished,’ ‘revealed,’ ‘reassembled,’ ‘reflexively,’ ‘reframed,’ ‘retrospectively,’ ‘repressed,’ ‘reference,’ ‘release,’ ‘reliquary,’ ‘remains,’ ‘research,’ and ‘retrace.’ Some of these terms we have used to describe Jarman’s and Morgan’s practices, and some we have used to describe our own critical practices.

Grouping together these terms may allow us to reflect on the relationship between the objects and activity of our research. For me that is one of the strands emerging from this dialogue: the acknowledged presence of ourselves as critics, which both the Jarman and Morgan archives introduced into the activity of research. When studying these reforgings of the medieval, in different ways we have both found ourselves engaging in less traditional modes of scholarship: more ‘inauthentic’ and/ or creative practices.

I was struck by your description of the challenge faced in studying these collections while being unable to photograph them. Institutional requirements enforce a concept of research as private, often through an intimidating legal language of copyright, permissions fees, breach of contracts, and so on. I found those institutional requirements difficult, speaking emotionally, when working on the Jarman collections. I wanted to photograph—reproduce—the objects so that I could share them with friends, and so that I didn’t have to be alone with these traces of the AIDS crisis. Since the Jarman archives are a public collection, anyone should be allowed to see them—but there’s no way of viewing the materials together as a community: we’d have to go in one by one and wait for each other in the lobby of the Tate, which would be a logistical nightmare and anyway wouldn’t create the experience of collective engagement. So that forces the researcher to be individualised.

It’s a huge privilege to have a private encounter with an artwork, in an archive that creates an intimacy of disclosure—I don’t want to downplay the romance of the academic lifestyle. But what we’re doing here in this dialogue piece is also a really freeing way to engage with materials, in a show-and-tell mode of scholarship that won’t necessarily lead us towards a fixed conclusion.

I’d like to turn to our own acts of remaking in more detail. During my project on Jarman, I had access to a research budget via the Wellcome/Birkbeck ISSF scheme that was also funding my short-term researcher’s salary, which I used to develop a collaborative essay-film response to my research: ‘submerged reliquary of a Kentish saint,’ made at the Derek Jarman Lab with director and artist Sophie Mei Birkin, and cinematographer Bartek Dziadosz. The film was screened at the Birkbeck Institute for the Moving Image in December 2022. Jarman’s description of the medieval past as a subterranean, jewelled reliquary was the epigraph of the film.

**Fig. 2:**
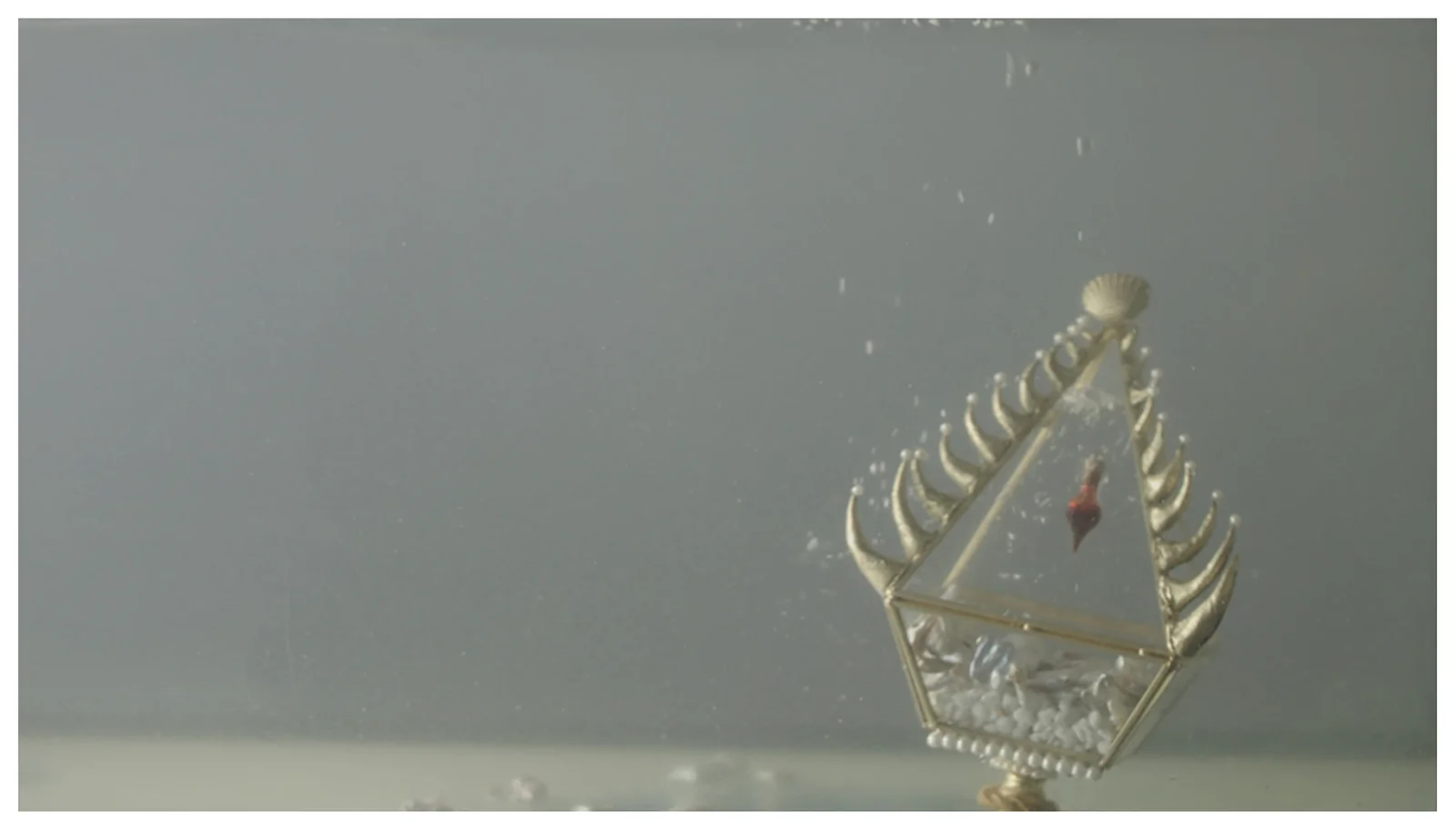
Reliquary, establishing shot. Sophie Mei Birkin, dir. ‘Submerged reliquary of a Kentish saint,’ 2022. 10 min. Screenshot from Vimeo.

**Fig. 3:**
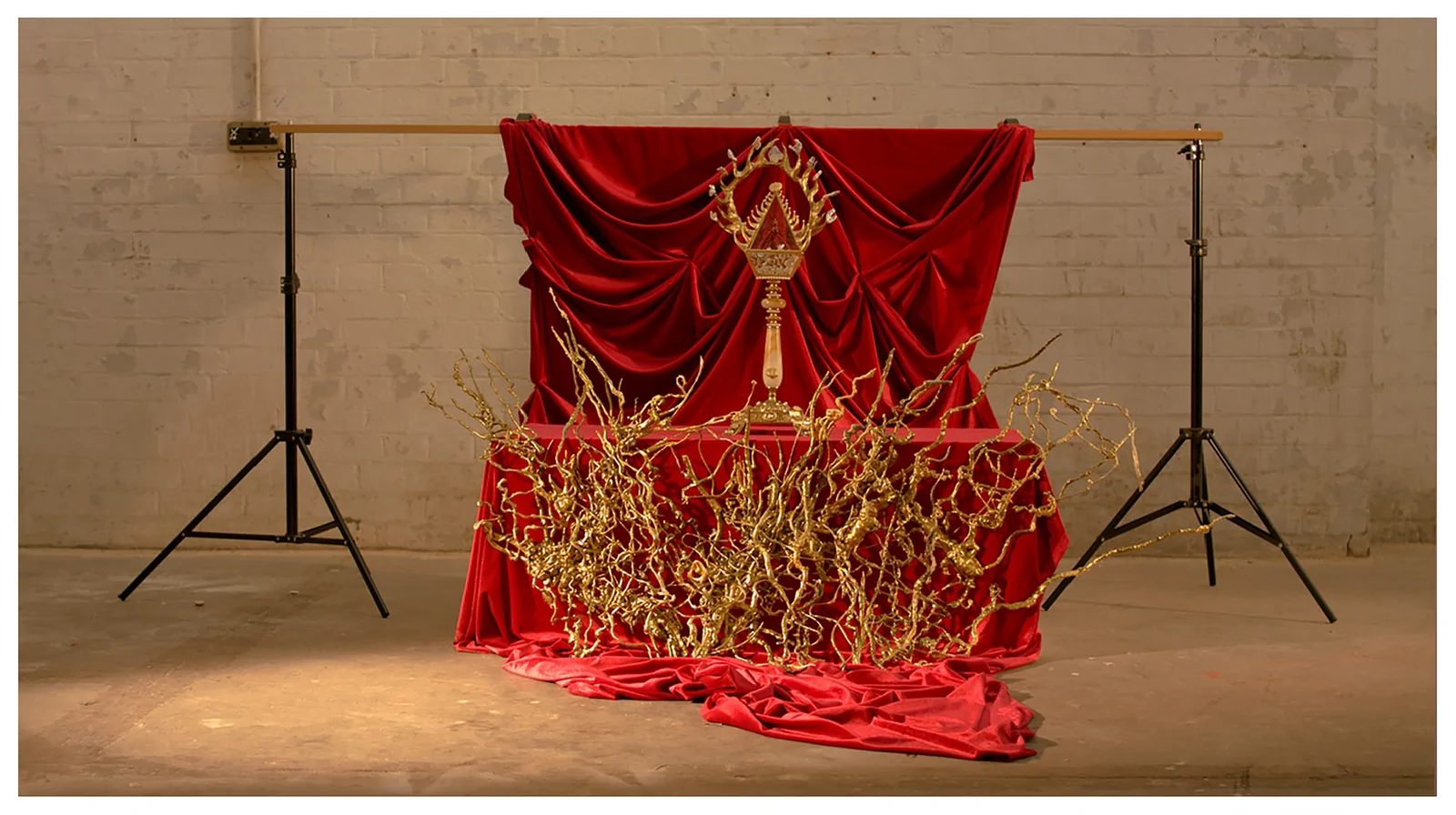
Falling reliquary. ‘Submerged reliquary of a Kentish saint.’ Screenshot from Vimeo.

**Fig. 4:**
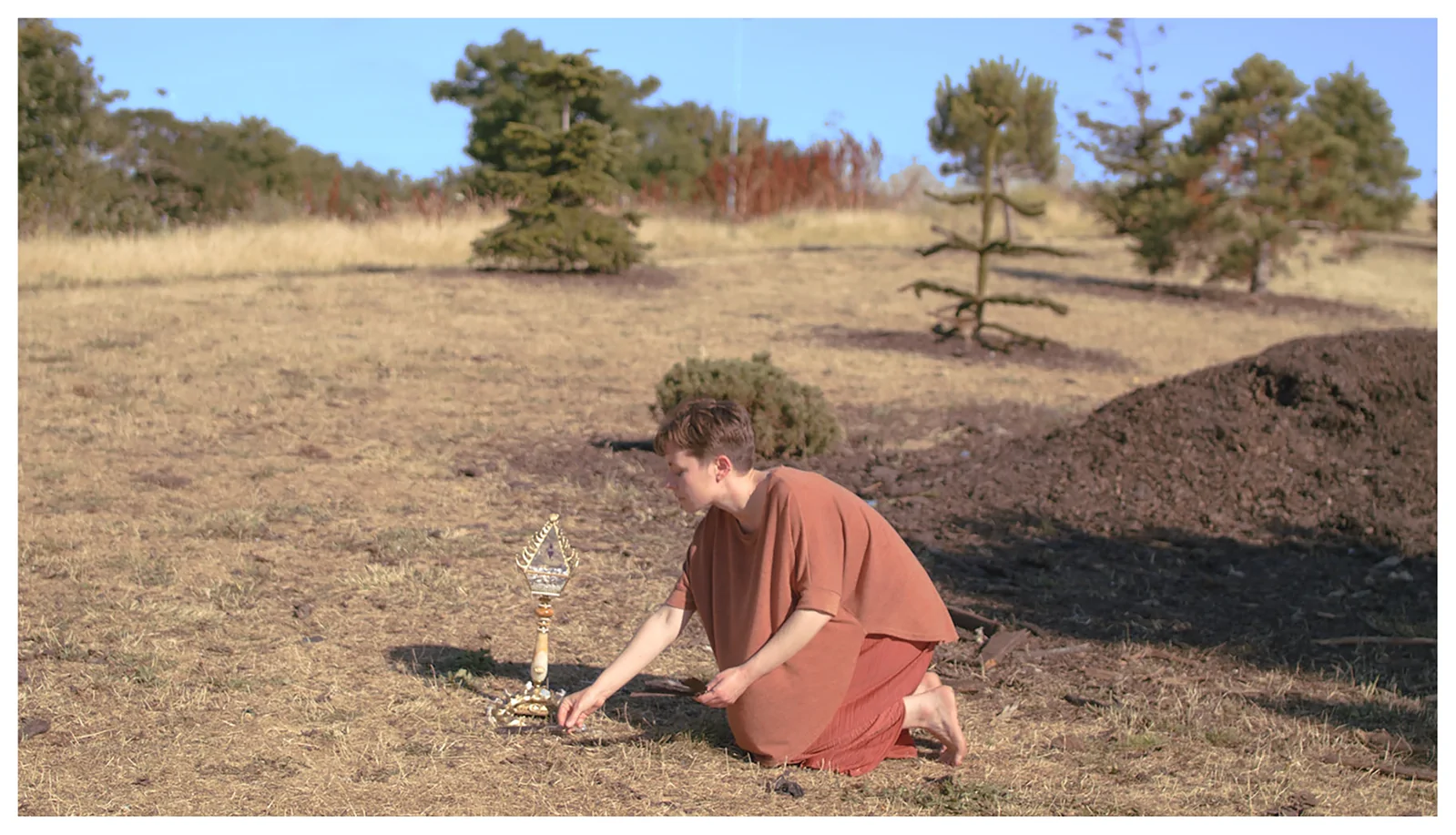
Ritual. ‘Submerged reliquary of a Kentish saint.’ Screenshot from Vimeo.

For the film, Sophie built a reliquary, which you can see here in three different contexts: on a stand (Fig. 2), submerged (Fig. 3), and in ritual use (by myself) (Fig. 4). The base of the reliquary is a Victorian table lamp bought on eBay, and the top is a terrarium. The seaweed-like branches are made from gold-painted foam. There is a vial of fake blood hanging inside the terrarium, and the reliquary is filled with freshwater seed pearls. The ‘crown’ also has pearls glued to it, as well as white beads, and one large opal. Like the archives that the reliquary aims to memorialise, the sculpture mixes precious and inexpensive materials.

I wrote a film script based on a journal that I kept around my trips to the archives, which was used as a voiceover: ‘Let us read together the life of St Derek of Dungeness and his holy deeds/and if I can I will take off these latex gloves and/touch.’ Creating our own inauthentic mourning ritual for Jarman felt like an engaged critical response to the materials that I’d encountered.

Sophie bought Super-8 film, the film which Jarman used for many of his early films. In the 1970s Super-8 was an inexpensive, amateurish medium, but it is now expensive to buy and to develop, so most of the footage was taken on a 90s camcorder—a technology popularised for home movies after Jarman’s death—and on a digital camera. Sophie experimented with using crystals and coloured gels, placed in front of the camera lens to act as prisms or otherwise alter the quality of the image—these are techniques which Jarman used. Seeing Sophie at work enabled me to write about both Jarman’s films and his journals with a more intimate sense of their making. In that sense, recreation was a direct kind of research—I wonder if you found the same when working with collage?

I want to think about performance as a mode of critical engagement with the past, both for Jarman and Morgan, and for ourselves as critics. For me, using a methodology in which I felt untrained and unqualified—I had zero prior experience working in film—brought me into a closer encounter with Jarman’s artworks. Dinshaw has written on the value of amateurism for medieval studies (2012, 37); both in terms of my analysis of Jarman’s medievalism and in my experience of filmmaking for research, I have to agree with her. This project was my first job after finishing my PhD in medieval literature, and there was something freeing about feeling like I was not even trying to use the methods that I’d gained through that qualification. As an amateur filmmaker (although I was being paid, so not really a typical amateur), I entered into my own rituals of submerging and alternative devotional practice. The process taught me something different about Jarman’s works, which would be harder to express in a traditional format. Would you be open to sharing an image of one of your collages with me? It feels fitting to discuss our own inauthentic/creative copies of the archives we’ve encountered, before (re-)turning to the Jarman and Morgan archives.

### Brooks

Thank you for this interesting turn in our conversation. It is fascinating and revealing to have our ‘re-’ terms catalogued like this and to reflect on the shared vocabulary we have been developing as we have passed this ‘show and tell’ of scholarship between ourselves. I’m now very aware that I have just used several ‘re-’ words in quick succession, but I wonder if we might add iterative and re-iterative to this list, to capture some of that sense of the fluidity of time, of the Middle Ages, and the act of circling back to time, text, and artefact. I am also very grateful to you here for turning our attention towards that question of the relationship between the ‘objects and activity of our research’ as you put it, and that sense of the tension between what can be made public from our research and what must remain private.

The stills from the film you made are so incredibly evocative, and I’m interested in the way Sophie Mei Birkin’s reliquary combines both precious (authentic?) and inauthentic materials to create this newly forged ritual artefact. As you say, this feels very appropriate for an homage to Saint Derek. Watching the film there is an almost dizzying sense of how the Jarman archive drew you into the intimate space of its disclosures. It is notable that your sense of the archive performing for you is translated here into a performance of your own: the performance of the archival researcher, putting on your mask and diving head-first into the submarine world of Jarman’s imagination. It is heady and beautiful and makes me think of Susan Howe’s much-quoted insight into working with the archive as a poet: ‘The inward ardor I feel while working in research libraries is intuitive. It’s a sense of self-identification and trust, or the granting of grace in an ordinary room, in a secular time’ (2014, 63). Sometimes we can’t deny our subjectivity in our encounter with the archive.

**Fig. 5:**
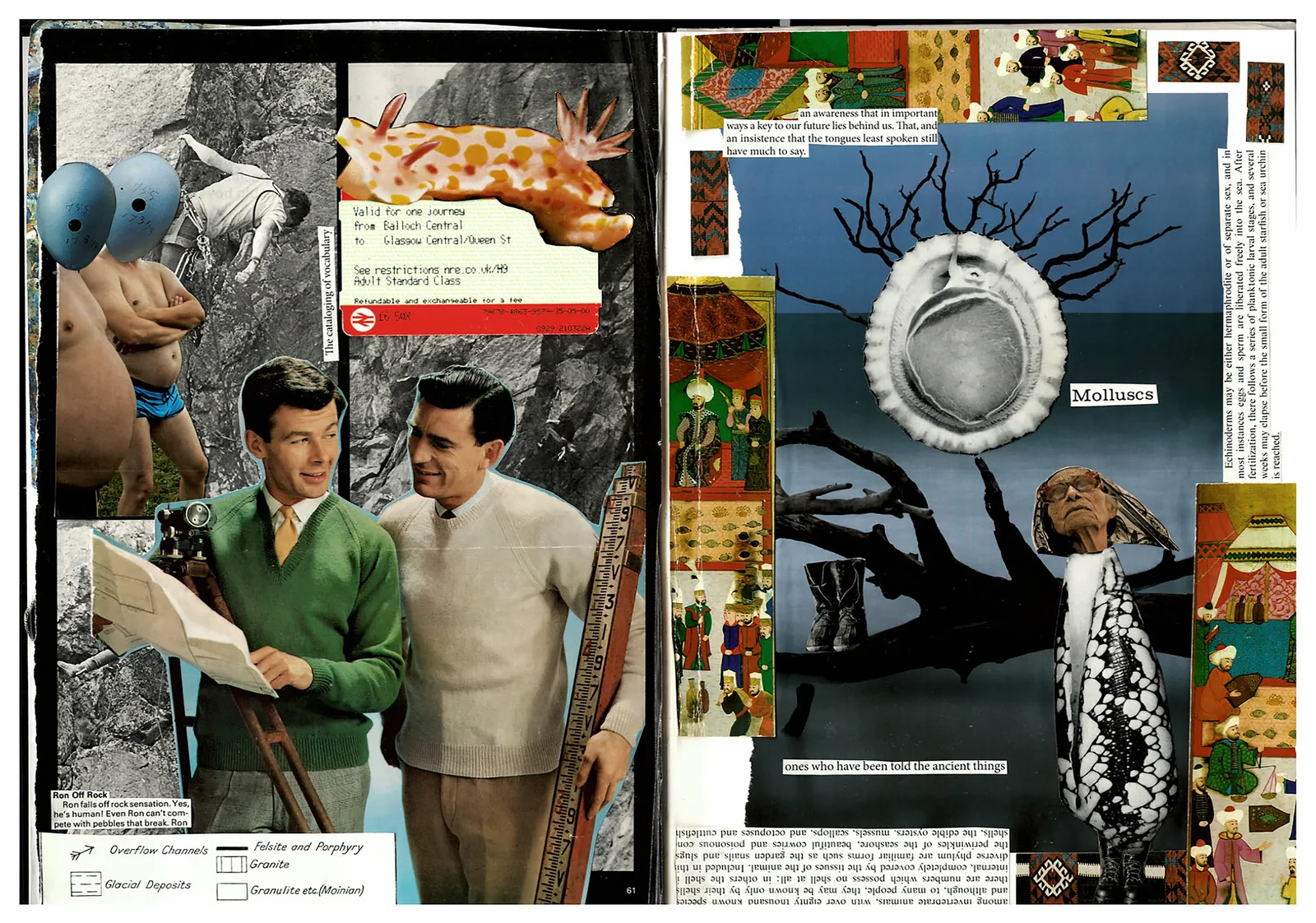
Collage page-spread from Francesca Brooks’s Glasgow Sketchbook. Image author’s own.

Here is one of the collages I created in response to Morgan’s scrapbooks (Fig. 5). Three others have been published in the 50th anniversary issue of the literary journal *From Glasgow to Saturn*, which asked for work inspired by Edwin Morgan, whose 1973 poetry collection gives the journal its name (Brooks 2023). As I have already suggested, Morgan’s scrapbooks were so unlike any other material, medieval or modern, that I had ever worked with before. Intellectually it was difficult to get my head around what I was working with, but aesthetically they were so inspiring and energising—it felt like I was getting a glimpse of Morgan’s poetic and artistic imagination in action, as if the pages were maps of influences and future poetic images, and now all I had to do was figure out how to read them. Something similar seems to be expressed in your script for the *Submerged Reliquary* film when you describe yourself as ‘like an archaeologist visiting a broken shrine.’ I wonder about the role our creative recreations have played when we have been confronted by shards and fragments of material in the archive that we are not quite able to make sense of as researchers. In my first week in the archive, I found myself itching to ‘have a go’ at scrapbooking myself. One evening I went to an art supply shop to pick up a sketchbook, pair of scissors, and some glue, and then I began collecting and hoarding materials that I might incorporate into my own Glasgow scrapbooks.

When I first began, I tried to replicate Morgan’s techniques quite directly and incorporated quotations from Morgan himself or things I had been reading that were informing my response to his scrapbooks. However, I soon became a bit freer in following my own impulses: the collages responded to the materials I found to work with as well as the experiences I was having in the archive, in the city of Glasgow, and on evening trips to museums and galleries, or on weekend day trips to Loch Lomond and the Ayrshire coast. There’s a chaotic treasure-trove of a bookshop in Glasgow on Otago Lane, Voltaire and Rousseau, and I went there to find disintegrating books I could salvage as collage material. The most significant of these finds was a book called *The Fringe of the Sea* by Isobel Bennett, which had formerly belonged to Glasgow Public Libraries. The book allowed me to respond to Morgan’s submarine interests with my own love of the coast and the deep sea. The images in it were so visually appealing that it became the centrepiece of many of the collages; this was something that I had recognised in Morgan’s scrapbooks too, where a series of images printed for an article on ‘Animals in Art’ came to dominate one early scrapbook visually and thematically.

I also picked up old mountaineering magazines, pamphlets about landscapes in Scotland, leaflets from exhibitions I had visited, train tickets, and wordlists in Gaelic. I think this gets at something that I have never found a satisfying way to articulate in my research, although I would like to: the fact that working with archives involves the kind of embodiment, and awareness of embodiment, that you have suggested, but also that it requires thinking over a sustained period in a place that might also be unfamiliar. That place, or just that time spent out-of-place on an archival trip, can also inform your thinking and become entangled with the intellectual work you do there.

I realised a few things about Morgan’s process through this act of ‘recreation,’ as you put it, as a direct kind of research. One realisation was about the sustained act of collection and curation that 3600 pages of a scrapbook require. Trying to dive into creating a scrapbook made me recognise that there is no shortcut to that kind of work, especially if it is going to reflect your own passions and thought process so vividly. This, in part, is why I ended up making collages rather than scrapbooks in a more Morgan-esque form: if I had wanted to include quotations, I would have had to collect them over a longer period and been willing to cut up a lot of books! Morgan’s Old English quotations all come, for example, from his copy of Henry Sweet’s *Anglo-Saxon Reader*, which he was using as an undergraduate. At some point he must have felt guilty about this act of vandalism because he tried to repair the gaps, creating what Chris Jones describes as a ‘Frankentext’ (2012, 133). The other realisation I had might seem like it is in tension with this awe at Morgan’s commitment to scrapbooking; the process also illustrated how much scrapbooking and collage relies on chance and impulse. Sometimes you plan for the inclusion of materials, but often something suggests itself intuitively and demands to be used. Although gathering materials involved planning and forethought, putting them together on the page was a much more spontaneous, creative act.

As we work towards a conclusion, I wonder if there is a final insight from the archive that you would like to share?

### Myerson

Thank you for sharing this collage. To start with an amateurish response, I love it—it is so witty and touching. I adore the queer intimacy of the two men in their button-downs, glancing at each other with their equipment, at once looking ready and totally unprepared for a journey—perhaps, implicitly, your journey from Balloch to Glasgow. Although I know some of your sources—mountaineering magazines, pattern leaflets, marine photographs—that knowledge does not demystify these juxtapositions. The ruler has ceased to be functional for measuring clothes: it has to be for mountains or the deep sea. The images have to float in their new environment. Looking at the image invites storytelling. Reading the line, ‘Even Ron can’t compete with pebbles,’ I immediately name one man as ‘Ron,’ looking over his lover’s shoulder and desperately wanting to be desired as much as the other man desires the ‘rock sensation’ of broken stones. Ron: in his new environs. Ron: in his moment. I tilt the page to read the line: ‘Echinoderms may be either hermaphrodite or of separate sex.’ This description of the fluid sex of the nonhuman reframes those who have entered the world of the collage: from the masked people with folded arms, to the composite non-person wearing the cool glasses on the right, their neck craned upwards from a chic-patterned shell/body and dykey boots: an aspirational ensemble. The central text could be the title of our dialogue: ‘Molluscs/ones who have been told the ancient things.’ The generative potential of molluscs and their ancient wisdom has been a subtheme: from the sea slugs in Morgan’s archives to the oysters that produced the freshwater pearls in the reliquary which Sophie Mei Birkin built.

I could riff off these images and texts in your collage for a lot longer, but I should go back to one last image of Jarman’s.

**Fig. 6:**
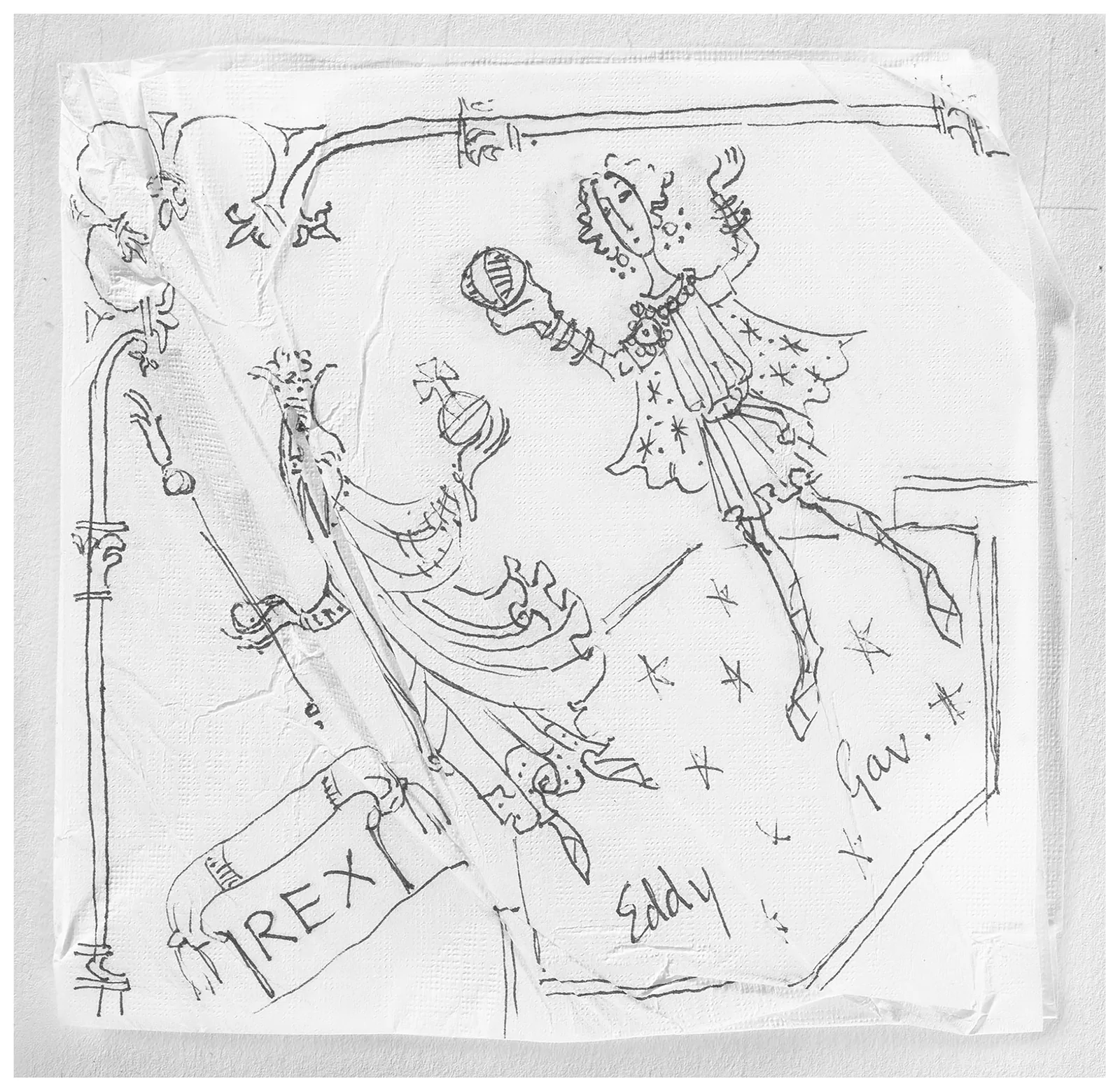
Tissue, Derek Jarman TGA 20213, Tate Britain. Photo © Tate. Courtesy Keith Collins Will Trust.

I found a tissue in the project book for Edward II (TGA 20213, 1990), stuck near the back of the sketchbook—on the tissue, is a sketch of ‘Eddy’ and ‘Gav’ (Fig. 6). The tissue is part of the intimate texture of the book. Jarman pasted it down, but at some point the tissue has been folded in such a way that to see the full image, the top layer of tissue has to be teased away from the page. When straightening the tissue to see the face of Queer King Eddy, which is obscured by the fold, I found that the men began to dance…. The movement of the tissue combined with the outstretched hands and flowing, star-specked cloak and tights to create a kind of disco in the archives. The dance was very brief as I could only pull out the tissue for a few seconds so as not to put too much pressure on the material—which feels like metonymy for the wider physical pressure which in-person archival work puts on researched materials. Part of the aim of digitisation is to prevent researchers from coming into physical archives too often. But the encounter which I had in person with the layers of this tissue allowed for a performance which cannot be reproduced with a 2D reproduction. Even the knowledge that I could easily tear the artwork gave a strange excitement to the process of stretching it out in my gloved hand.

To go back to our discussion of ‘re-’ terms, I completely agree with your additions of ‘iterative’ and ‘re-iterative.’ Putting Jarman’s archives together with medieval archives, I had a nonlinear experience of time in that research process. I had previously researched medieval texts in dialogue with contemporary artworks, but analysing artworks on public display or in reproduction—the experience of touching the recent past—was new to me. When working with these medieval and modern archives at the same time, the 1990s felt stranger, more fragile, and more distant to me than the 1390s.

Jarman’s archives memorialise contemporary queers as well as the premodern, remembering both: re-membering, putting queer bodies back together through the language of the past.

### Brooks

The tissue offers a perfect image of the archive performing for you in a moment of embodied, intimate research. I am struck again by the space this dialogue has provided to reflect on research experiences that are sometimes hard to account for in our public or academic writing.

I have written in quite broad terms about the thematic concerns of Morgan’s scrapbooks and in minute terms about how a few lines of a translation might mimic the visual processes of scrapbooking and collage. It seems appropriate now to turn to one page in a little more detail, which is from the seventh scrapbook and annotated with a date-span of 1946–1953. When this scrapbook was wheeled out, I was immediately struck by how different its physicality was to the other books. Scrapbook 7 is huge and incredibly heavy; the scale of it is ostentatious—it made me think of medieval gospel books, and this physical association was clearly intentional and one that Morgan played with. C/7 is dominated by visual and verbal imagery from devotional texts and religious paintings. Other sources muse on death, the afterlife, and hell, and as this is a post-war scrapbook it also reflects on the devastations and lasting impact of the war, from Nazi atrocities to the atomic bombings of Hiroshima and Nagasaki. Early medieval culture and literature resurface in this scrap-book, having lain dormant in some previous books, and the Old English quotations incorporated throughout take us from creation (*Genesis A* and *B*) to Judgement Day (*Judgement Day II*).

**Fig. 7:**
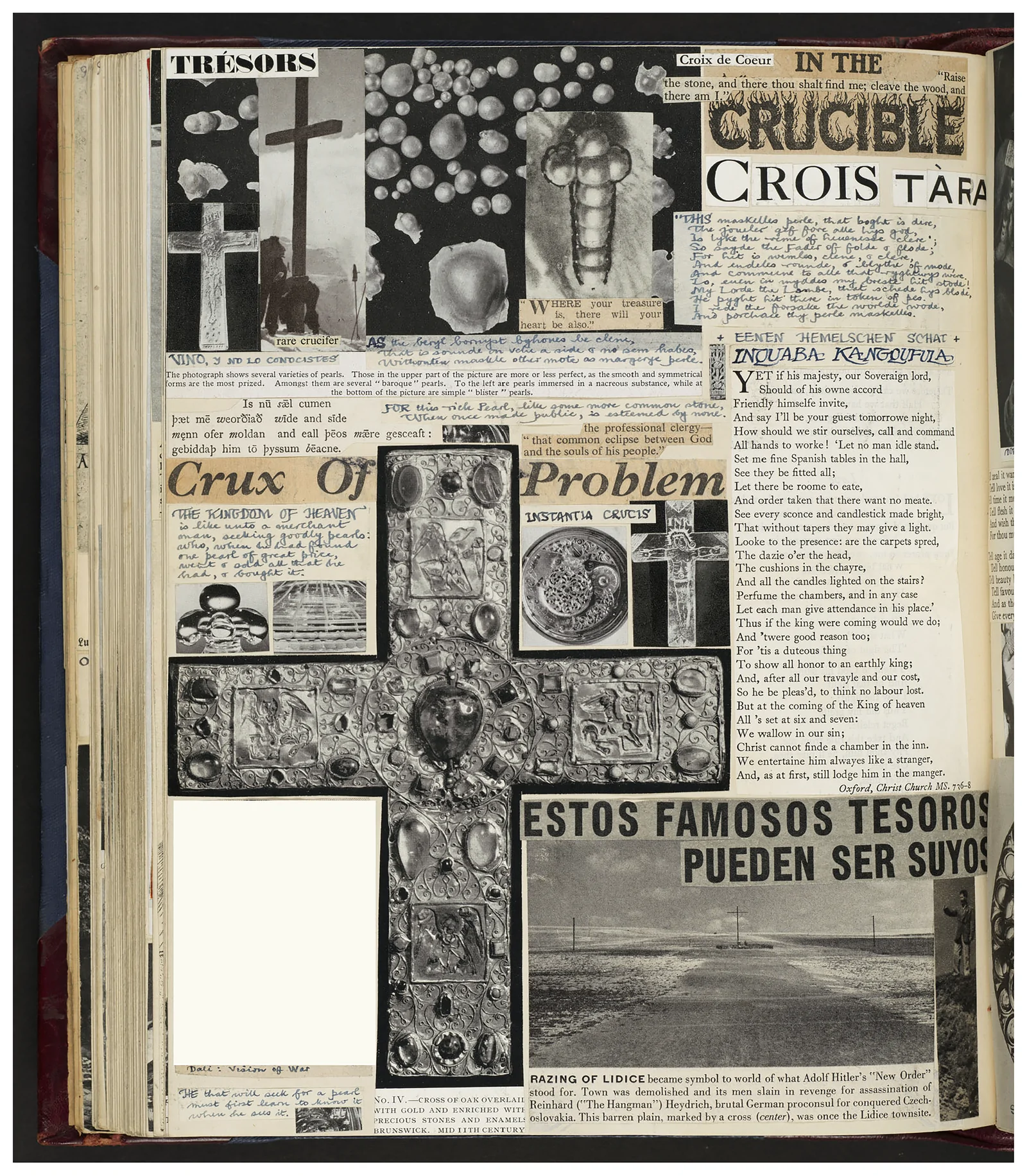
MS Morgan C/7, Single page from Scrapbook 7, 1059, University of Glasgow Library Archives and Special Collections, with Salvador Dali’s painting *The Visage of War* blanked out because of copyright restrictions. Photo © University of Glasgow Library Archives and Special Collections. Courtesy of the Edwin Morgan Trust and the University of Glasgow Library.

The page I want to talk about (MS Morgan C/7, 1059) presents a multilingual concrete poem and meditation on the cross in a time of war (Fig. 7). The collage layers images of pearls, crosses, and a highly ornate *crux gemmata* (an eleventh-century ceremonial cross commissioned by Countess Gertrude), with ‘cross’ wordplay, and quotations including from the Old English *Dream of the Rood* and the Middle English poems *Pearl* and *Cleanness*. If we read the page vertically we can see it as taking us from the heavenly heights of the ‘maskeles perle’ to the hellish underworld where Salvador Dali’s tortured painting *The Visage of War* speaks to the horror of a photograph of a solitary cross marking the site of the Lidice massacre, a town in what is now the Czech Republic that was razed to the ground in 1942 at the order of Adolf Hitler and Heinrich Himmler. There are several readings we might draw out from this page. Is the ‘crux of the problem’ (as one pasted headline reads) that religious symbols have been emptied of their meaning and resonance in this period of human atrocity? Should we see this emphasis on ‘treasure’—the Spanish phrase reads ‘these famous treasures could be yours’—as tinged with irony in the face of such destruction? Or is this an attempt to find some consolation in these enduring symbols of faith and revelation? Is the page an act of prayer or devotion that tries to imagine more hopeful, renewed futures? Ultimately scrapbooking and collage are highly ambiguous media that create space for holding and exploring uncertainty through processes of decontextualisation and juxtaposition (see Brinkman 2011; Cran 2014). Although the visual and textual material of the medieval past encountered in the prismatic space of Morgan’s scrapbooks is as alien and mysterious as the ‘stark pelagic potsherds’ of Montale’s poems, their translation is essential to the vivid language Morgan created to meet the future.

## Conclusion

These themes of pearls and underwater exploration recall Hannah Arendt’s description of Walter Benjamin keeping ‘little notebooks […] in which he tirelessly entered in the form of quotations what daily living and reading netted him in the way of “pearls”’ (1968, 200). Morgan and Jarman’s scrapbooks share this practice of collection. At times the search for pearls was literal: both artists sought out molluscs and their sacred products. Like deep water, at times these collections feel heavy, as with Morgan’s large-scale Scrapbook 7, and at times they give an illusion of weightlessness, as with the tissue in Jarman’s project book for Edward II. These are physical experiences: it is difficult for digital images to produce the same affect, the same sensation of floating and sinking in the archives. In their open, fluid form, these collections invite researchers to submerge themselves.

What might come from this process of submersion? And how does it relate to our critical and creative impulses as researchers? In *Material Thinking*: *The Theory and Practice of Creative Research* Paul Carter writes:

If research involves finding something that was not there before, it ought to be obvious that it involves imagination. If it is claimed that what is found was always there (and merely lost), still an act of creative remembering occurs. As a method of materialising ideas, research is unavoidably creative. (2004, 7)

Is it possible to engage in research without participating in this act of creative remembering? As researchers we often go to the archive in the hopes of authenticating certain intuitions we have about the genesis of the literary and visual works we are studying, but is authenticity ever quite possible in a performative space that demands and invites our own affective response as readers? Thinking through how Morgan and Jarman creatively translated and reforged the material of the Middle Ages has made us both more aware of the role that imagination plays in our research. Perhaps imagination sounds like an unlikely tool for a pair of medievalists who must also be attentive to precise forms of knowledge—contextual and linguistic, for example—but, as Clare A. Lees and Gillian Overing affirm, ‘creative practices in the present’ can restore much ‘to our thinking about the past’ (2019, 4–5). Both Jarman and Morgan deployed historic medieval practices of creation and reception, inviting us to reconsider the past on its own terms. The queer contents of their archives allow us to re-enter the medieval not merely as an object to be considered at a distance, but as a space in which we can submerge ourselves, to reemerge, reforged.
